# Peripheral Endogenous Cannabinoid Levels Are Increased in Schizophrenia Patients Evaluated in a Psychiatric Emergency Setting

**DOI:** 10.3389/fpsyt.2020.00628

**Published:** 2020-06-30

**Authors:** Stéphane Potvin, Louiza Mahrouche, Roxane Assaf, Marjolaine Chicoine, Charles-Édouard Giguère, Alexandra Furtos, Roger Godbout

**Affiliations:** ^1^ Department of Psychiatry, Centre de recherche de l’Institut Universitaire en Santé Mentale de Montréal, Montreal, QC, Canada; ^2^ Department of Psychiatry, University of Montreal, Montreal, QC, Canada; ^3^ Department of Chemistry, University of Montreal, Montreal, QC, Canada; ^4^ Sleep Laboratory and Clinic, CIUSSS du Nord-de-l’Île-de-Montréal, Hôpital en santé mentale Rivière-des-Prairies, Montréal, QC, Canada

**Keywords:** anandamide, oleoylethanolamide, schizophrenia, emergency setting, cannabinoids

## Abstract

**Background:**

The endogenous cannabinoid system mediates the psychoactive effects of cannabis in the brain. It has been argued that this system may play a key role in the pathophysiology of schizophrenia. While some studies have consistently shown that the levels of anandamide, an endogenous cannabinoid ligand, are increased in the cerebrospinal fluid of schizophrenia patients, inconsistent results have been observed in studies measuring anandamide levels in the periphery. Here, we sought to determine if the assessment of peripheral anandamide levels in patients evaluated in a psychiatric emergency setting would show robust increases.

**Methods:**

One hundred seven patients with a schizophrenia-spectrum disorder from the psychiatric emergency settings of the *Institut Universitaire en Santé Mentale de Montréal* and 36 healthy volunteers were included in the study. A subsample of thirty patients were assessed at two time points: at the emergency and at their discharge from the hospital. Anxious and depressive symptoms, sleep and substance use were assessed using self-report questionnaires. In addition to anandamide, the levels of oleoylethanolamide (OEA), an anorexigenic fatty-acid ethanolamide, were also measured, since the prevalence of the metabolic syndrome is increased in schizophrenia. Plasma levels of anandamide and OEA were measured using liquid chromatography and mass spectrometry.

**Results:**

Plasma anandamide and OEA levels were significantly increased in schizophrenia patients, relative to controls (Cohen’s d=1.0 and 0.5, respectively). Between-group differences remained significant after controlling for metabolic measures. No differences were observed between schizophrenia patients with and without a comorbid substance use disorder at baseline. Importantly, the levels of both endocannabinoids significantly decreased after discharge from the emergency setting.

**Conclusion:**

The current results add to the growing body of evidence of endocannabinoid alterations in schizophrenia. The strong elevation of plasma anandamide levels in schizophrenia patients assessed in the psychiatric emergency setting suggests that anandamide and OEA area potential biomarkers of the psychological turmoil associated with this context.

## Introduction

Schizophrenia is a complex psychiatric disorder, and its pathophysiology is not fully understood. During the last decades, several longitudinal studies have shown that cannabis smoking is a risk factor for psychosis outcomes (1). In adult populations with schizophrenia, several studies have also shown that persistent cannabis smoking is associated with worse outcomes (2). Moreover, several experimental studies have shown that the administration of delta-9-tetrahydrocannabinol to healthy volunteers produces transient effects that are similar to the psychiatric symptoms and cognitive deficits seen in schizophrenia (3, 4). These findings have fueled interest in examining the potential role of the endogenous cannabinoid system, which mediates the psychoactive effects of cannabis in the brain, in the pathophysiology of schizophrenia (5, 6).

The endogenous cannabinoid (ECB) system is complex and is composed of two primary natural ligands, namely anandamide and 2-arachidonoylglycerol (2-AG), and two primary receptors, CB_1_ and CB_2_ (7). Virodhamine, N-arachidonoyl-dopamine and noladin ether are increasingly considered as ECB ligands (8), and vanilloid receptor 1 and GPR55 as potential ECB receptors (9, 10); however, their precise roles remain to be determined. Anandamide is synthesized from N-acetylphosphatidyl-ethanolamine (NAPE) by NAPE-hydrolysing phospholipidase D and degraded by fatty acid amid hydrolase (FAAH) (11) into ethanolamine and arachidonic acid (12). 2-AG is synthesized from diacylglycerol (DAG) by DAG lipase and degraded by monoacylglycerol lipase into glycerol and arachidonic acid (13). Interestingly, anandamide is involved in key functions that are known to be altered in schizophrenia, including reward processing, stress regulation and memory (14, 15). Moreover, CB_1_ receptors are distributed in high densities in brain regions known to be impaired in schizophrenia, such as the prefrontal cortex, the hippocampus and the basal ganglia (7, 16).

Preliminary evidence suggests that the ECB system is involved in the pathophysiology of schizophrenia. Indeed, *postmortem* human brain studies using auto radiography have consistently shown that CB_1_ receptor binding is elevated in the dorso-lateral prefrontal cortex in schizophrenia (6). The *postmortem* studies on CB_1_ receptor mRNA levels in the dorso-lateral prefrontal cortex have produced mixed results however (17, 18). As for *in vivo* studies, a recent positron emission tomography (PET) study has shown an increase in CB_1_ receptor binding in 67 schizophrenia patients in several brain regions, including the ventral striatum, the insula, the inferior frontal cortex and the medial temporal cortex (19). Likewise, Wong et al. (20) had also observed an increase in CB_1_ receptor binding in the pons in a small sample of 9 schizophrenia patients. However, a more recent PET study actually showed a decrease in CB_1_ receptor binding in several sub-cortical and limbic regions (21). Regarding the ECB ligands, an elevation of anandamide levels in the cerebrospinal fluid (CSF) of schizophrenia patients was initially reported by Leweke et al. (22) in 10 schizophrenia patients (22). Subsequently, CSF anandamide levels were found to be eight-fold higher in 47 schizophrenia patients than in 84 healthy controls and individuals with other psychiatric disorders (23). Importantly, the finding of elevated CSF anandamide levels in schizophrenia has been replicated since then (24). CSF anandamide levels were also found to be elevated during the initial prodromal stages of psychosis (25).

Due to the ease of measurement, a growing number of laboratories have examined peripheral levels of endogenous cannabinoids in schizophrenia, with results being inconsistent across studies thus far. In a study of 20 schizophrenia patients, the blood levels of anandamide were shown to be higher in patients with acute schizophrenia compared to healthy controls (26). Similarly, Koethe et al. (25) found that plasma levels of anandamide are elevated in twins discordant for schizophrenia compared to healthy twins (25). Furthermore, the expression of CNR1, the gene coding for the CB_1_ receptor, was found to be up-regulated in the peripheral blood of schizophrenia patients (27). Despite these promising results, other studies looking at blood levels of anandamide did not detect any differences between schizophrenia and healthy controls (24, 28).

The heterogeneity of findings on peripheral levels of anandamide in schizophrenia could be explained by different factors. One important factor is the phase of illness. In the acute phase of illness or during emergency visits, when patients are experiencing significant stress, anandamide levels may be more increased. In fact, it has been shown in experimental studies performed in healthy volunteers that acute stress provokes increases in peripheral anandamide levels (29). Thus far, two studies have been performed in schizophrenia patients during the acute phase of illness—at least to our knowledge. A first study showed significantly higher levels of anandamide in patients compared to healthy controls (26), although it only included a small sample of 12 patients. However, a study from Giuffrida et al. (23) showed no significant alterations of serum anandamide levels in acutely paranoid schizophrenia patients.

As argued by Desfossés et al. (30), important comorbid factors such as substance use and metabolic problems may also influence results. Substance use disorders are highly prevalent in schizophrenia and are associated with poorer clinical outcomes in this population (31). In a dually diagnosed population, our research team found that plasma anandamide levels were increased, relative to controls, and that there was a positive correlation between anandamide levels at baseline and substance use at 3-month follow-up (32). As for the metabolic syndrome, its prevalence is two to three times higher in patients with schizophrenia (30-40% prevalence) than in the general population (10-20%) (33). Given that anandamide is involved in food control intake *via* central and peripheral mechanisms (34), we performed a pilot functional neuroimaging study and showed that plasma levels of anandamide were positively correlated with amygdala hyper-activations in schizophrenia patients in response to appetizing food stimuli (35). In addition, an association has been observed between the CNR1 gene and the metabolic syndrome in 407 patients with schizophrenia (36). Although structurally related to anandamide, oleoylethanolamide (OEA) is a non-cannabinoid natural bioactive fatty-acid ethanolamide, which binds peroxisome-proliferator-activated receptors, and is degraded by FAAH into oleic acid and ethanolamide (37). OEA has well-demonstrated anorexic properties (37). In schizophrenia, most studies on CSF and blood levels of OEA have shown no significant alterations (22, 23, 25). However, it is crucial to point out that these studies have not accounted for comorbid metabolic problems.

The primary objective of the current study is to show that peripheral levels of anandamide are increased in schizophrenia patients evaluated in a psychiatric emergency setting. The secondary objective is to examine the clinical correlates of anandamide and OEA levels in schizophrenia.

## Methods

### Participants

**Figure 1:**
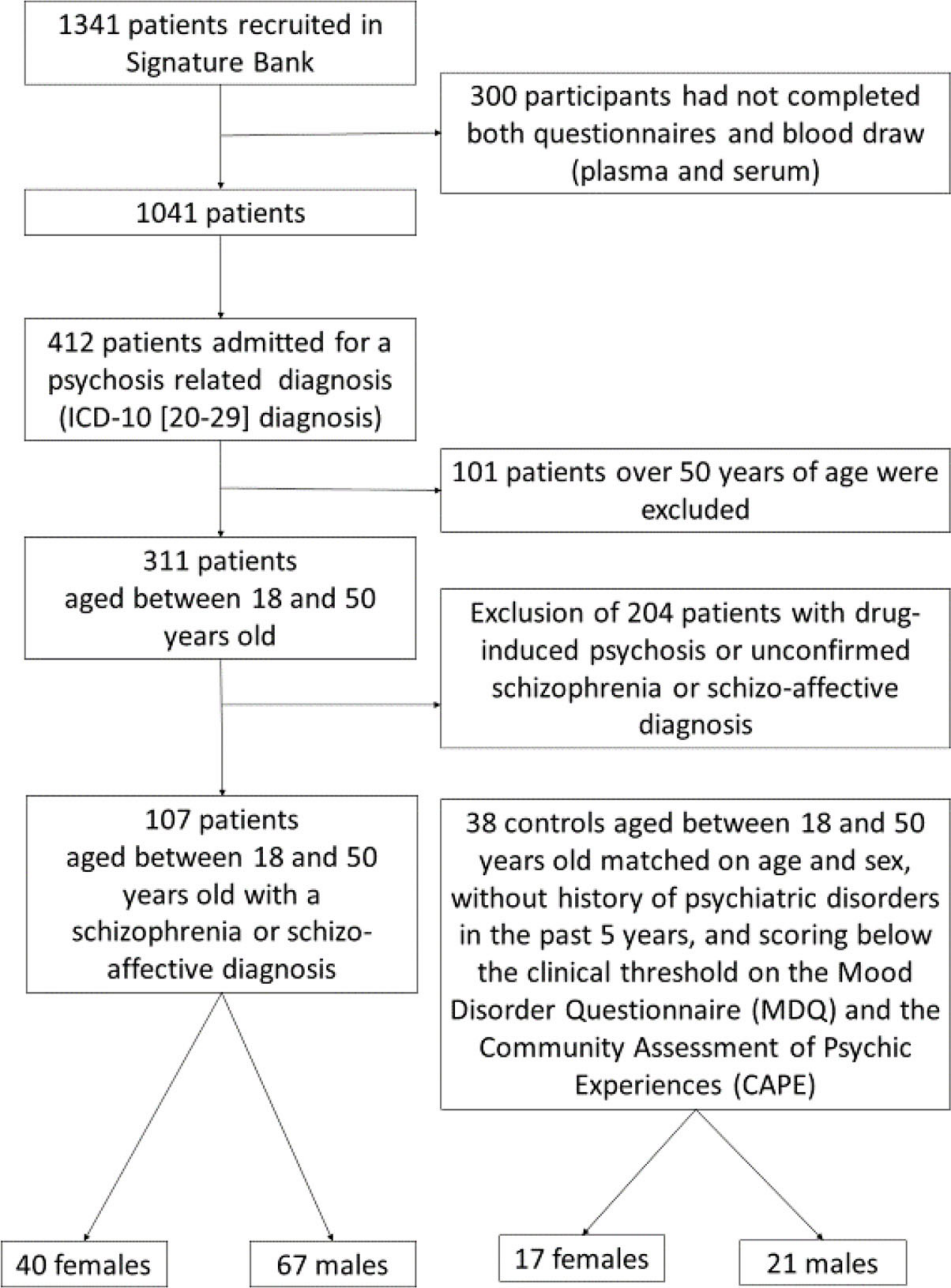
Flowchart of the participants sample selection.

One hundred seven patients with schizophrenia or schizo-affective disorder, male or female, and aged between 18 and 50 years old, were recruited at the psychiatric urgency setting of the *Institut Universitaire en Santé Mentale de Montréal*. The sample was taken from the Signature Bank of the institute (http://www.iusmm.ca/recherche/signature.html). Patients enrolled in the Signature Bank were referred to the research team by the clinical emergency team (nurses, social worker, and emergency psychiatrist), who met each morning to discuss every new case. Psychiatric diagnoses were established by psychiatrists on the ward, and were coded according to the *World Health Organisation International Classification of Disease*, ICD-10 (38). Diagnoses were confirmed after psychiatric hospitalization. The mean number of psychiatric hospitalizations in the last two years was 2.5 (± 3.4), and 34.7% of patients experienced their first episode of psychosis. In this naturalistic study, substance use disorder and metabolic syndrome were not considered as exclusion criteria in the schizophrenia group. Out of 107 schizophrenia patients, 14 had a current comorbid substance use disorder (alcohol, cannabis and/or stimulants). Patients with substance-induced psychosis were however excluded if a schizophrenia of schizo-affective disorder was not confirmed by the psychiatrist. **Figure 1** shows the flowchart for the selection of the 108 patients. Schizophrenia patients were treated with antipsychotics (mean olanzapine equivalents: 13.0 ± 11.6 mg); among them, 22 were treated with two antipsychotics or more, and 10 were treated with clozapine. Thirty-eight healthy volunteers, with no history of severe mental illness or substance use disorder, were also recruited. None of the healthy controls were treated with medication affecting the central nervous system. Both groups did not differ in terms of age (schizophrenia: 31.5 ± 8.2 years; controls: 30.0 ± 7.3 years; t=1.0; p=0.32) and sex ratio (schizophrenia: 40 females; controls: 17 females; χ^2^ = 0.36; p=0.55). None of the participants in either group had a history of neurologic disorder, an IQ lower than 70, or chronic and unstable medical diseases at the moment of participation in the study. A subsample of thirty patients had measures at two time points: at the emergency (T1; emergency phase) and at their discharge from the hospital (T2; stabilization phase). Patients were discharged on clinical decision resulting in a variable duration of admission. At baseline, the subsample of 30 patients did not differ from the rest of patients in terms of socio-demographic variables, psychiatric symptoms and metabolic markers (see **Supplementary Table**).

All participants signed a detailed consent form, and the study was approved by the ethics committee of the *Centre de Recherche de l’Institut Universitaire en Santé Mentale de Montréal*.

### Clinical Assessments

Substance use disorder severity was assessed with the *Alcohol Use Disorders Identification Test* (AUDIT) (39) and the *Drug Abuse Screening Test* (DAST-10) (40). Psychotic symptoms, depressive symptoms and anxiety were measured, respectively, with the *Psychosis Screening Questionnaire* (PSQ) (41), the *Patient Health Questionnaire* (PHQ-9) (42), and the *State Trait Anxiety Inventory* (STAI) (43); sleep problems were assessed with a validated questionnaire from our team (44). The potential influence of antipsychotics on results was examined by calculating olanzapine equivalents (45).

### Metabolic Syndrome

A 12-h fasting blood collection (38 ml) was obtained in the morning for the emergency patients and healthy controls. Within 2 h, the local hospital laboratory assayed serum total cholesterol, high-density lipoprotein, low-density lipoprotein, triglycerides, and fasting glucose, using standard hospital techniques. Resting seated systolic and diastolic blood pressure and heart rate was measured. Anthropometric measures (e.g. body mass index and waist-to-hip ratio) were also collected.

Metabolic syndrome was defined as the presence of 3 or more of the risk factors identified by the *International Diabetes Federation*: (i) *waist circumference*: males≥ 102 cm; females≥ 88 cm; (ii) *triglycerides*≥ 150 mg/dl; (iii) *high-density lipoprotein* (HDL): males≤ 40 mg/dl; females≤ 50 mg/dl; (iv) *arterial pressure*: systolic pressure≥ 130 mmHg; diastolic pressure≥ 85 mmHg; and (v) *fasting glucose*≥ 100 mg/dl) (46).

### Analysis of Plasma Anandamide (AEA) and OEA Levels

We collected blood samples (10 ml) of participants in the morning after 12 h of fasting. Within 2 h, blood samples were centrifuged (2600 rpm for 15 min), and plasma (1 ml) was stored at −80°C in glass vials. Calibration curve standards were prepared in a pooled human plasma (Innovative research, Novi, MI) using standards of AEA-d4 and OEA-d4 (Cayman Chemical, Ann Arbour, MI) ranging from 0.02 ng/ml to 6 ng/ml and were kept frozen. Freshly thawed plasma aliquots and calibration curve standards (450 μL) were diluted with 900 μL of cold acetonitrile containing 10 ng/ml of the internal standard, AEA-d8 (Cayman Chemical, Ann Arbour, MI). Samples were then loaded to an Impact protein precipitation plate from (Phenomenex, Terrance, CA). The flow through was diluted with 500 μL HPLC grade water and submitted to solid phase extraction on Hydrophilic-Lipophilic Balance Oasis HLB 30 mg cartridges from (Waters). Eluted compounds were dried down under a nitrogen stream and reconstituted in 75 μL of the starting mobile phase. Aliquots of 15 μL were injected into the liquid chromatography-mass spectrometry (LC-MS) system. Chromatography was performed on an 1100 series from Agilent Technologies (Santa Clara, CA) using a Charged Surface Hybrid C18, 2.1x100 mm, 3.5 μm column from Waters (Milford, MA). The eluents consisted of 40% acetonitrile and 60% water (solvent A) and 90% isopropanol and 10% acetonitrile (solvent B), both containing 0.4% formic acid. The initial mobile phase contained 35% B and was increased to 45% B over 10 min. Endocannabinoids were monitored on a triple quadrupole mass spectrometer 6410 from Agilent Technologies (Santa Clara, CA) operated in positive Electrospray Ionization using the Multiple Reaction Monitoring mode. The LC-MS method was linear for both AEA and OEA from 0.02 to 6 ng/ml. Samples were run on four batches, over four days. Coefficients of Variation as measured for the Quality Controls across the four days were within 9.6 % for AEA and 7.4 % for OEA.

### Statistical Analyses

Potential group differences for dichotomic variables and continuous variables were examined, respectively, with chi-square tests and two-sample *t* tests. The potential relationships between endocannabinoid levels and clinical variables (e.g. psychiatric symptoms, sleep and metabolic variables) were examined using linear regression analyses. For the regression analyses, the potential association between endocannabinoid levels and metabolic variables was examined by calculating the number of metabolic syndrome criteria met by each participant. Finally, a subsample of patients (n=30) were assessed at admission (T1) and at their discharge (T2). A paired *t* test was assessed to check for changes between T1 and T2. Pearson’s correlation tests were performed to assess if antipsychotic dosage (in olanzapine equivalents) was associated with each of the endocannabinoid levels. Statistical analyses were performed with R version 3.6.3. The threshold for statistical significance was set at p < 0.05. For each series of variables, a false-discovery rate (FDR) was applied to the p-value to account for type-II error. Both corrected and uncorrected p-values are presented.

## Results

### Between-Group Differences

**Figure 2:**
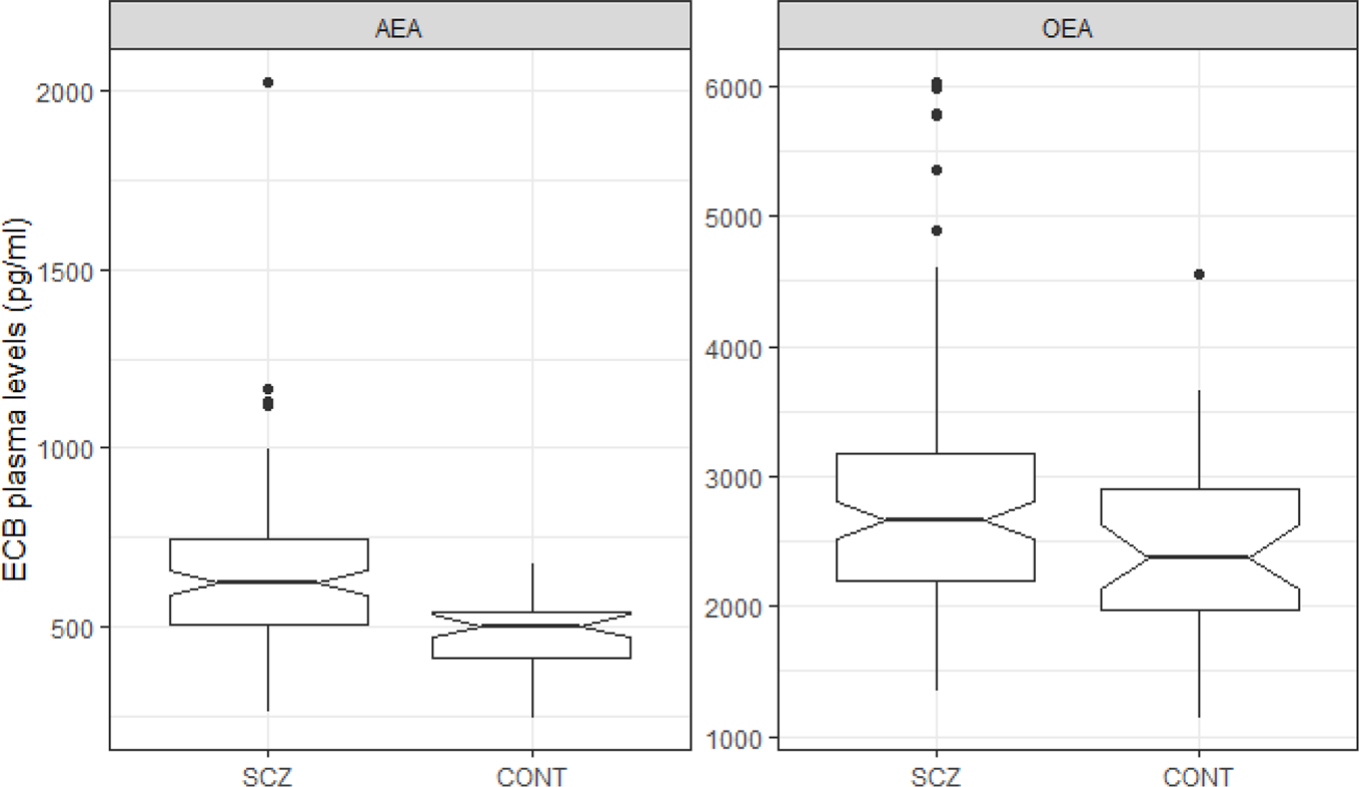
Boxplot of ECB plasma levels by Group (Schizophrenia vs. Controls).

Relative to controls, schizophrenia patients had increased psychiatric symptoms (e.g. anxiety, depression, psychosis and poor sleep efficiency), as well as higher scores on substance use scales (e.g. AUDIT and DAST) (**Table 1**). A higher proportion of patients presented the metabolic syndrome, compared to controls (schizophrenia: 34.0%; controls: 8.1%; χ^2^ = 7.99; p=0.005). Furthermore, metabolic markers (e.g. Waist circumference, Triglyceride, HDL) were found to be impaired in schizophrenia. More importantly, anandamide levels were strongly increased in schizophrenia patients, relative to controls, with a large effect size (Cohen’s d=0.9; p < 0.001) (**Table 1** and **Figure 2**). OEA levels differed between patients and controls with a smaller effect-size (Cohen’s d=0.5; p=0.013) (**Table 1** and **Figure 2**). Between-group differences in anandamide and OEA levels remained significant after controlling for the potential influence of metabolic markers. Likewise, there were no differences in ECB levels between patients with and without a substance use disorder (anandamide: p=0.56; OEA: p=0.43).

**Table 1:** Differences between schizophrenia patients and healthy controls.

Type of variable	Variable	Schizophrenia (n=107)	Controls (n=38)	Statistics
Endogenous cannabinoids	Anandamide	653.7 (222.8)	469.8 (110.1)	t=6.5; p < 0.001
	OEA	2856.8 (969.7)	2416.2 (716.7)	t=2.9; p=0.005
Psychiatric symptoms	AUDIT	5.2 (6.9)	4.1 (2.8)	t=1.3; p=0.19
	DAST	3.1 (3.2)	0.5 (0.6)	t=8.0; p < 0.001
	Psychosis	1.5 (1.4)	0.0 (0.0)	t=11.1; p < 0.001
	Anxiety	47.8 (14.8)	34.3 (10.2)	t=6.2; p < 0.001
	Depression	10.6 (7.0)	2.6 (3.1)	t=9.4; p < 0.001
Sleep	Sleep efficiency (%)	91.7 (16.0)	96.4 (7.9)	t=-2.3; p=0.025
Metabolic Syndrome	Waist circumference (cm)	97.2 (18.1)	88.2 (18.9)	t=2.5; p=0.013
	Triglyceride (mg/dl)	1.6 (1.2)	0.9 (0.5)	t=4.6; p < 0.001
	HDL (mmol/L)	1.1 (0.3)	1.4 (0.3)	t=-6.1; p < 0.001
	Mean arterial pressure (mmHg)	90.4 (11.0)	89.6 (9.7)	t=0.4; p=0.66
	Glycemia (mmol/L)	5.1 (1.0)	4.9 (0.5)	t=1.4; p=0.16
	Number of metabolic syndrome indicators	1.9 (1.3)	0.8 (1.2)	t=4.6; p < 0.001

AUDIT, Alcohol Use Disorder Identification Test; DAST, Drug Abuse Screening Test; HDL, high-density lipoprotein; OEA, oleoylethanolamide; the metabolic syndrome is obtained when the number of syndrome indicators is three or more.

### Regression Analyses

**Figure 3:**
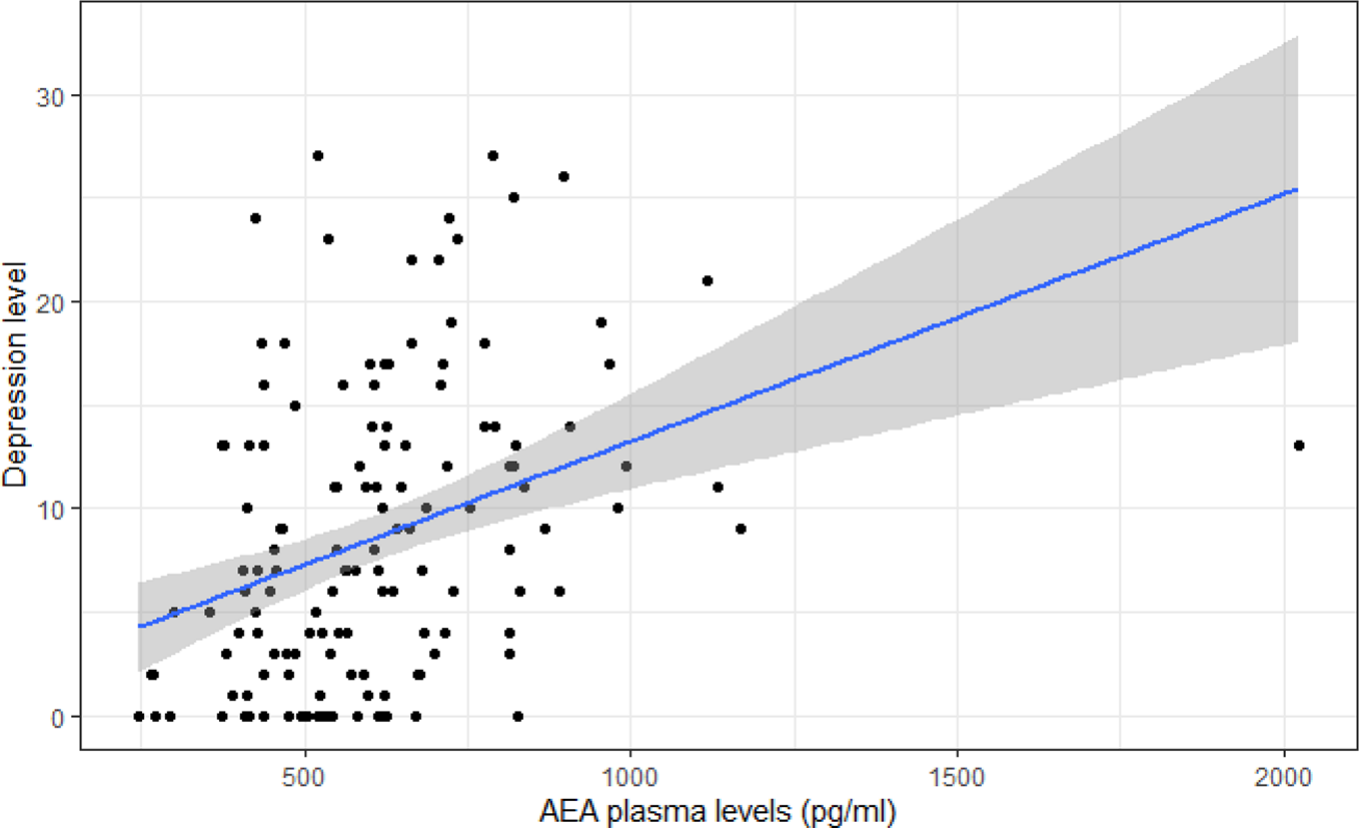
Dispersion plot of depression levels (PHQ-9) on AEA levels.

Across groups, a significant and positive association was found between anandamide levels and depressive symptoms (p=0.009) (**Table 2** and **Figure 3**). This association remained significant after adjusting for the false-discovery rate (p^*^=0.043). A smaller negative association was found between anandamide levels and sleep efficiency (p=0.053) (**Table 2**). Across groups, there were no significant correlations between endogenous cannabinoid levels and substance use, anxiety and psychosis (**Table 2**). Positive associations of small magnitude were observed between anandamide levels and waist circumference (p=0.013) and arterial pressure (p=0.018), while a small negative association was found between OEA levels and triglyceride levels (p=0.038) (**Table 2**). These associations were no longer significant after accounting for the FDR adjustment. No association was found between the number of metabolic syndrome indicators and ECB levels.

**Table 2:** Linear regression analyses.

Clinical variable		Anandamide					OEA				
		Est.	SE	t	p	p^*^	Est.	SE	t	p	p^*^
AUDIT	Int.	2.642	1.787	1.478	0.14	0.14	3.491	1.725	2.024	0.045	0.045
	Group	-0.269	1.270	-0.212	0.83	0.83	-0.725	1.210	-0.599	0.55	0.55
	ECB	0.004	0.003	1.521	0.13	0.28	0.001	0.001	1.058	0.29	0.63
DAST	Int.	2.855	0.817	3.493	<0.001	0.001	2.668	0.784	3.402	<0.001	<0.001
	Group	-2.542	0.580	-4.381	<0.001	<0.001	-2.542	0.550	-4.621	<0.001	<0.001
	ECB	0.000	0.001	0.288	0.77	0.77	0.000	0.000	0.555	0.58	0.63
Psychosis (PSQ)	Int.	1.672	0.347	4.821	<0.001	<0.001	1.637	0.334	4.899	<0.001	<0.001
	Group	-1.539	0.247	-6.238	<0.001	<0.001	-1.509	0.234	-6.456	<0.001	<0.001
	ECB	-0.000	0.000	-0.571	0.57	0.71	-0.000	0.000	-0.483	0.63	0.63
Anxiety (STAI)	Int.	42.579	4.003	10.637	<0.001	<0.001	45.479	3.884	11.71	<0.001	<0.001
	Group	-12.456	2.857	-4.359	<0.001	<0.001	-13.569	2.726	-4.979	<0.001	<0.001
	ECB	0.008	0.006	1.388	0.17	0.28	0.001	0.001	0.642	0.52	0.63
Depression (PHQ-9)	Int.	6.095	1.789	3.407	<0.001	0.001	7.481	1.746	4.285	<0.001	<0.001
	Group	-6.715	1.277	-5.259	<0.001	<0.001	-7.503	1.226	-6.120	<0.001	<0.001
	ECB	0.007	0.003	2.668	**0.009**	**0.043**	0.001	0.001	1.901	0.06	0.30
Sleep efficiency (%)	Int.	99.408	4.179	23.786	<0.001	<0.001	93.817	4.086	22.96	<0.001	<0.001
	Group	2.552	3.019	0.845	0.40	0.40	4.426	2.900	1.526	0.13	0.13
	ECB	-0.012	0.006	-1.950	0.053	0.053	-0.001	0.001	-0.544	0.59	0.59
Waist circumference (cm)	Int.	84.780	5.254	16.135	<0.001	<0.001	97.320	5.183	18.78	<0.001	<0.001
	Group	-5.318	3.751	-1.418	0.16	0.24	-8.814	3.640	-2.421	0.017	0.025
	ECB	0.019	0.008	2.506	**0.013**	0.054	-0.000	0.002	-0.026	0.98	0.98
Triglyceride (mg/dL)	Int.	1.950	0.304	6.420	<0.001	<0.001	2.144	0.292	7.356	<0.001	<0.001
	Group	-0.739	0.219	-3.371	<0.001	0.002	-0.712	0.205	-3.472	<0.001	0.001
	ECB	-0.001	0.000	-1.325	0.19	0.28	-0.000	0.000	-2.097	**0.038**	0.23
HDL (mmol/L)	Int.	1.146	0.083	13.863	<0.001	<0.001	0.955	0.079	12.021	<0.001	<0.001
	Group	0.335	0.060	5.625	<0.001	<0.001	0.372	0.056	6.651	<0.001	<0.001
	ECB	-0.000	0.000	-0.816	0.42	0.50	0.000	0.000	1.706	0.09	0.27
Mean arterial pressure (mmHg)	Int.	83.477	3.074	27.156	<0.001	<0.001	86.851	3.009	28.86	<0.001	<0.001
	Group	0.917	2.194	0.418	0.68	0.68	-0.485	2.112	-0.230	0.82	0.82
	ECB	0.011	0.004	2.393	**0.018**	0.054	0.001	0.001	1.261	0.21	0.42
Glucose (mmol/L)	Int.	5.117	0.263	19.451	<0.001	<0.001	5.249	0.254	20.64	<0.001	<0.001
	Group	-0.185	0.190	-0.972	0.33	0.40	-0.196	0.179	-1.094	0.28	0.33
	ECB	-0.000	0.000	-0.215	0.83	0.83	-0.000	0.000	-0.777	0.44	0.53
# of metabolic syndrome indicators (0-5)	Int.	1.426	0.377	3.789	<0.001	<0.001	2.203	0.366	6.017	<0.001	<0.001
	Group	-0.941	0.272	-3.463	<0.001	0.002	-1.119	0.258	-4.341	<0.001	<0.001
	ECB	0.001	0.001	1.350	0.18	0.28	-0.000	0.000	-0.865	0.39	0.53

AUDIT, Alcohol Use Disorder Identification Test; DAST, Drug Abuse Screening Test; ECB, endocannabinoid; HDL, high-density lipoproteins; OEA, oleoylethanolamide; PHQ-9, Patient Health Questionnaire-9; PSQ, Psychosis Screening Questionnaire; STAI, State Trait Anxiety Inventory; SE, standard error; p*= p-value adjusted for the false-discovery rate.

### Paired t Test

The mean length of hospitalization was 26.1 days (± 22.4). For both endocannabinoids, the mean levels decreased between admission (T1) and discharge (T2). In the case of anandamide, we observed a mean decrease of 132.6 pg/ml (p < 0.001) and as for OEA, a decrease of 639.7 pg/ml (p=0.005) (**Table 3**). Aside from endocannabinoids, four clinical outcomes showed significant improvements from T1 to T2, namely psychosis (0.024), anxiety (0.013), depression (p < 0.001), and mean arterial pressure (0.05) (**Table 3**).

**Table 3:** Paired t-test to assess the differences between endocannabinoid levels and clinical variables at admission (T1) and at release (T2).

Endocannabinoids (pg/ml; n=30)	Mean (sd) T1	Mean (sd) T2	Mean Diff. (T1-T2)	t	p	p^*^
Anandamide	543.5 (171)	410.8 (139)	132.6	3.76	<0.001	0.002
OEA	2287.0 (915)	1647.3 (757)	639.7	3.07	0.005	0.005
AUDIT	4.9 (7.5)	4.6 (7.8)	0.30	0.53	0.60	0.60
DAST	3.0 (3.1)	2.5 (2.8)	0.57	1.32	0.20	0.25
Psychosis (PSQ)	1.5 (1.4)	0.9 (1.0)	0.53	2.39	0.024	0.040
Anxiety (STAI)	45.3(15.0)	38.4 (15.0)	6.89	2.64	0.013	0.033
Depression (PHQ-9)	10.2 (6.2)	6.0 (4.9)	4.23	4.35	<0.001	<0.001
Sleep efficiency (%)	95.4 (8.3)	97.7 (4.1)	-2.30	-1.38	0.18	0.18
Waist circumference (cm)	97.0 (13.5)	98.5 (12.5)	-0.91	-0.78	0.44	0.84
Triglyceride (mg/dL)	1.6 (1.1)	1.6 (1.0)	0.02	0.10	0.92	0.92
HDL (mmol/L)	1.1 (0.3)	1.1 (0.3)	0.01	0.38	0.71	0.85
Mean arterial pressure (mmHg)	93.5 (11.0)	89.2 (8.9)	4.32	2.05	0.050	0.30
Glycemia (mmol/L)	5.1 (0.9)	5.3 (2.0)	-0.16	-0.58	0.56	0.84
# of metabolic syndrome indicators (0-5)	1.8 (1.2)	1.6 (1.5)	0.27	1.35	0.19	0.57

AUDIT, Alcohol Use Disorder Identification Test; DAST, Drug Abuse Screening Test; ECB, endocannabinoid; HDL, high-density lipoproteins; OEA, oleoylethanolamide; PHQ-9, Patient Health Questionnaire-9; PSQ, Psychosis Screening Questionnaire; STAI, State Trait Anxiety Inventory. p*= p-value adjusted for the false-discovery rate.

### Antipsychotic Treatment

No statistically significant associations were found between prescribed antipsychotic dosage (e.g. olanzapine equivalents) and plasma ECB levels (rAEA: r=0.06; p=0.58; OEA: r= −0.02; p=0.89). Between-group differences in anandamide and OEA levels remained significant after controlling for antipsychotic dosage.

## Discussion

Despite substantial evidence that cannabis is a risk factor for psychosis (1) and growing evidence suggesting that the endocannabinoid system is altered in schizophrenia, studies examining peripheral levels of anandamide have produced inconsistent results thus far. In the current study, we sought to determine the blood levels of anandamide and OEA in schizophrenia patients recruited at the emergency setting. As hypothesized, we found that anandamide levels were robustly increased in patients relative to a group of healthy volunteers; in the case of OEA, there was also an increase at baseline but it was smaller. Importantly, we observed a significant decrease in both biomarkers in a subset of patients after discharge from the emergency setting. As such, these results suggest that anandamide, and OEA to a lesser extent, are potential biomarkers of the stress induced by an acute mental crisis prior to the presentation to the emergency department. In addition, we observed a positive correlation between peripheral levels of anandamide and depressive symptoms. However, there was no association with psychotic symptoms, unlike the previous reports of negative correlations between psychotic symptoms and anandamide levels, as measured in the CSF (23) and the serum (47). Being in a psychiatric inpatient setting is a source of significant stress for patients with schizophrenia, and as mentioned in the introduction, acute stress has been shown to result in increased peripheral anandamide levels in healthy volunteers (29). Moreover, it is well documented that stress is a risk factor for depression (48–50) and that the hypothalamic-pituitary-adrenal (HPA) axis is disturbed in major depressive disorder (51). Taken together, these observations suggest that anandamide alterations are more related to emotional turmoil associated with the psychiatric emergency setting rather than the severity of psychotic symptoms. At the physiological level, this association could be mediated by a dysregulation of the HPA axis.

A secondary objective of the current study was to examine the potential association of peripheral ECB levels and common comorbidities (e.g. substance use disorder and metabolic syndrome) in schizophrenia. In the current study, the prevalence of the metabolic syndrome was increased in schizophrenia patients, relative to controls, consistently with the vast literature on the topic (33). Moreover, small associations were found between metabolic variables and plasma anandamide and OEA levels, which were no longer significant after applying corrections for multiple comparisons. In animal studies, there is strong evidence showing that CB_1_ receptor agonists and OEA exert control over food intake *via* central and peripheral mechanisms, including hepatic triglyceride biosynthesis (34, 37). In humans, mounting clinical evidence gathered in populations with no severe mental illness suggests that blood levels of anandamide and OEA are increased in obese individuals (52, 53). Moreover, complex associations between plasma OEA levels and limbic activity (e.g. insula) elicited by food cues have been observed in obese and control individuals (54). In addition, controlled trials of the CB_1_ inverse agonist rimonabant for the treatment of obesity have shown significant reductions in body weight, triglyceride levels and the prevalence of the metabolic syndrome (55). Similarly, preliminary evidence suggests that OEA reduces appetite in obese people (56). As mentioned in the introduction, preliminary evidence has linked the ECB system to appetite dysregulation in schizophrenia (35, 36). Overall, the significant associations between anandamide and OEA and metabolic variables are consistent with current evidence. Finally, we found a small and negative relationship between anandamide and sleep efficiency. To our knowledge, this is the first study describing such an association in schizophrenia. The result is consistent with the increasing evidence on anandamide as a sleep regulator (57).

Unexpectedly, we found no relationships between ECBs (anandamide and OEA) and substance use problems. In the past, two studies have examined the influence of substance use on ECB levels in schizophrenia. Our team found that plasma anandamide and OEA levels were increased in a population of schizophrenia patients with comorbid substance use disorder (mainly alcohol and cannabis) (32). Conversely, another team found that CSF anandamide levels were increased in schizophrenia patients who used cannabis *occasionally*, relative to controls, whereas there were no differences in CSF anandamide levels between controls and schizophrenia patients who used cannabis *frequently* (58). Considering that we found no association, here, between substance use severity and plasma levels of ECBS, the available evidence suggest that the impact of substance use on ECBs in schizophrenia is complex, and that results are influenced by factors such as the pattern of substance use (e.g. use, frequent use, disorder), the type of substance (e.g. cannabis and/or alcohol) and the biological sample used to measure ECBs (e.g. blood versus CSF).

The current study has a few limitations that need to be acknowledged. First, the positive symptoms (delusions, hallucinations) of schizophrenia patients were assessed with the PSQ, a self-report instrument, although interview-based assessments are considered as the gold standard in the field (59). Due to a lack of insight, patients may not self-report psychotic symptoms. This may not only impede the assessment of whether patients are in the acute phase of illness or not, but also impede the investigation of a potential association between ECB levels and psychotic symptoms. Second, schizophrenia patients were treated with antipsychotic medication before blood collection. Although the impact of antipsychotics on peripheral anandamide and OEA levels are currently poorly understood (32, 35), we cannot rule out this confounding effect. However, no significant associations were found between antipsychotic dosage and ECB plasma levels. Moreover, it is worth noting that psychiatric hospitalization is associated with very poor drug compliance in schizophrenia (60). On the other hand, the main strength of the current study is that it investigated peripheral anandamide and OEA levels in the largest sample of schizophrenia patients assessed in the psychiatric emergency setting (at least, to our knowledge), and that a subgroup of these patients were reassessed after release from the emergency when they no longer had acute symptoms.

The results of the current study show that plasma anandamide and OEA levels are significantly increased in schizophrenia patients evaluated in the psychiatric emergency setting. As such, anandamide and OEA are candidate biomarker of this phase. In the future, longitudinal studies will need to be performed in larger samples of schizophrenia patients in both the acute phase of illness and after psychiatric stabilization. Future studies will also need to assess a larger range of endocannabinoid biomarkers that are not restricted to anandamide and OEA, and to examine if changes in ECBs vary according to antipsychotic response. Finally, the potential interactions between ECBs and the HPA axis will need to be investigated in schizophrenia.

## Data Availability Statement

The datasets presented in this article are not readily available because The current study was performed using an institutional databank including genetic information, and the ethics committee has not granted permission to make the dataset of individual studies available to the community. Requests to access the datasets should be directed to stephane.guay.CEMTL@ssss.gouv.qc.ca.

## Ethics Statement

The studies involving human participants were reviewed and approved by Centre de Recherche de l’Institut Universitaire en Santé Mentale de Montréal. The patients/participants provided their written informed consent to participate in this study.

## Author Contributions

LM and AF performed the biochemical analyses. RG, MC, and SP designed the study. SP and RG provided funding. Statistical analyses were performed by C-EG. SP wrote the manuscript. All authors provided critical comments.

## Funding****

The study was funded by Bell Canada mental health initiatives, Centre de recherche de l’Hôpital Rivière-des-Prairies, the Institut Universitaire en Santé Mentale de Montréal and a grant from the Canadian Institute of Health Research to SP. 

## Conflict of Interest

The authors declare that the research was conducted in the absence of any commercial or financial relationships that could be construed as a potential conflict of interest.
